# Distinct Functional Connectivity Patterns Are Associated With Social and Cognitive Lifestyle Factors: Pathways to Cognitive Reserve

**DOI:** 10.3389/fnagi.2019.00310

**Published:** 2019-11-13

**Authors:** Jessica I. Fleck, Molly Arnold, Benjamin Dykstra, Katharine Casario, Elizabeth Douglas, Otto Morris

**Affiliations:** School of Social and Behavioral Sciences, Stockton University, Galloway, NJ, United States

**Keywords:** cognitive reserve, resting-state EEG, lagged linear connectivity, cognitive function, gender

## Abstract

The importance of diverse lifestyle factors in sustaining cognition during aging and delaying the onset of decline in Alzheimer’s disease and related dementias cannot be overstated. We explored the influence of cognitive, social, and physical lifestyle factors on resting-state lagged linear connectivity (LLC) in high-density electroencephalography (EEG) in adults, ages 35–75 years. Diverse lifestyle factors build cognitive reserve (CR), protecting cognition in the presence of physical brain decline. Differences in LLC were examined between high- and low-CR groups formed using cognitive, social, and exercise lifestyle factors. LLC is a measure of lagged coherence that excludes zero phase contributions and limits the effects of volume conduction on connectivity estimates. Significant differences in LLC were identified for cognitive and social factors, but not exercise. Participants high in social CR possessed greater local and long-range connectivity in theta and low alpha for eyes-open and eyes-closed recording conditions. In contrast, participants high in cognitive CR exhibited greater eyes-closed long-range connectivity between the occipital lobe and other cortical regions in low alpha. Greater eyes-closed local LLC in delta was also present in men high in cognitive CR. Cognitive factor scores correlated with sustained attention, whereas social factors scores correlated with spatial working memory. Gender was a significant covariate in our analyses, with women displaying higher local and long-range LLC in low beta. Our findings support distinct relationships between CR and LLC, as well as CR and cognitive function for cognitive and social subcomponents. These patterns reflect the importance of diverse lifestyle factors in building CR.

## Introduction

Cognitive reserve (CR) is an individual’s ability to preserve cognitive function, in the presence of a decline in the physical brain due to age, injury, or disease, such as in Alzheimer’s disease (Stern, 2002, 2009). It has been suggested that engaging in cognitive, social, and physical activities throughout the lifespan may build high levels of CR (e.g., Scarmeas et al., 2003; Dekhtyar et al., 2015; Clare et al., 2017), offering the individual protection against the sequelae of physical brain decline. Although the benefits of CR on cognition in older adults has been established, the neural mechanisms that enable a declining brain to produce normal cognition are less clear. By examining CR-related differences in connectivity within and among cortical regions, our research begins to quantify the importance of diverse lifestyle factors in explaining the range of cognitive function that is possible during aging.

To explore the impact of CR on cognitive function, researchers and clinicians use an individual’s participation in stimulating lifestyle factors to estimate probable CR level (Farina et al., 2018; Stern et al., 2018). Implementing CR proxies is necessary because the real measurement of CR is only possible through the direct assessment of physical brain health and concurrent cognitive function (see Stern et al., 2018). Historically, verbal IQ, years of formal education, and occupational complexity were applied as CR proxies in research (Stern, 2006; Jones et al., 2011). More recently, social sengagement (Evans et al., 2018; Sauter et al., 2019), leisure activities (Yates et al., 2016), and exercise (Ji et al., 2018; Reas et al., 2019) have been emphasized as components that may build CR. The expansion of CR proxies is essential to enable an accurate estimate of CR in diverse participant groups (see Jones et al., 2011). However, the distinct and combined contributions of various lifestyle factors to CR is not well understood.

CR may alter the impact of age-related brain changes on cognitive function, thus mitigating the influence of physical decline on cognition (for a review, see Stern et al., 2018). It has consistently been observed that high-CR patients with Mild Cognitive Impairment (MCI) and Alzheimer’s disease (AD) exhibit higher levels of brain pathology than low-CR patients with similar cognitive function, reflecting the ability of high-CR patients to support cognition in the face of more pronounced physical brain decline (e.g., Pettigrew et al., 2017; Soldan et al., 2017). Additionally, research on healthy older adults has reported that participants with high CR exhibit lower task-related brain activation than participants with low CR, suggestive of greater network efficiency in individuals with higher CR (Scarmeas et al., 2003; Speer and Soldan, 2015). From these findings, it has been suggested that CR supports neural efficiency during healthy aging and compensatory processing in clinical decline (Bosch et al., 2010).

CR level has been shown to moderate the relationship between resting-state brain activity and cognitive function in healthy older adults and patient groups. Resting-state brain activity reflects activation in brain networks when the individual is awake and relaxed but is not engaged in task-directed cognition (for a review, see van den Heuvel and Hulshoff Pol, 2010). Activity in the resting-state is an important metric of brain network function with broad clinical applications and utility, due to its comprehensive assessment of brain network function and ease of use with diverse populations (e.g., AD, schizophrenia, young children; Rosazza and Minati, 2011; Stam, 2014). Using fMRI, Bastin et al. (2012) observed lower activity in resting-state networks in participants who were high in CR, which may indicate improved network efficiency in high CR individuals. In patient populations, Franzmeier et al. (2017) reported that MCI patients with high CR possessed more voxels with high global connectivity in the brain’s cognitive control network, whereas MCI patients with low CR possessed more voxels with high local connectivity (see also Serra et al., 2017) Most recently, Lee et al. (2019) reported positive correlations between CR level and global network efficiency, as well as local connectivity, in healthy-adult, MCI, and AD participant groups. These findings suggest that the organization of resting-state brain networks is associated with CR level and reflect the influence of CR on brain network organization.

Preliminary research has identified relationships between CR and cortical connectivity measured using electroencephalography (EEG). EEG captures electrophysiological changes in the brain with millisecond resolution, making it ideal to assess the coordination of brain regions (He et al., 2011). During prior research in our lab, we observed distinct patterns of cortical connectivity in resting-state EEG for high- vs. low-CR participants in younger vs. older sample members (Fleck et al., 2017). In younger members of our sample (<58 years of age), participants with the highest CR levels displayed the lowest global coherence levels, particularly in theta and gamma frequencies, whereas older members of the sample who were high in CR exhibited greater global coherence than low-CR participants across frequency bands. CR was estimated using years of formal education in conjunction with verbal IQ scores. For both age groups, high-CR participants exhibited better performance on measures of cognitive function than low-CR participants. Most recently, Sanchez-Lopez et al. (2018) reported better performance on the subtests of the WAIS IQ test, along with increased alpha and decreased delta and theta power in participants who reported high levels of incidental exercise (e.g., yard work and housework). Taken together, these findings suggest that resting-state EEG is sensitive to the electrophysiological changes associated with various CR proxies.

The primary goal of the present research was to determine the relationships between cognitive, social, and physical CR factors and brain connectivity, during resting-state EEG. Additionally, we examined the relationships among different CR factors and cognitive function. All participants had their resting-state brain activity recorded with high-density EEG and completed a general cognitive battery, as well as questionnaires to assess their participation in various CR-related lifestyle factors. Care was taken to assess a range of lifestyle factors in cognitive, social, and physical domains. Brain connectivity was measured within and between brain regions to estimate local and long-range connectivity. Because of the importance of age and gender in brain aging, it has been suggested that men’s brains age faster than women’s brains (Király et al., 2016; Goyal et al., 2019), we included age and gender as covariates in our design.

We used lagged linear connectivity (LLC) as our measure of the connectivity among cortical regions of interest. LLC is a measure of the linear relationship between two signals, excluding instantaneous coherence which can be affected by volume conduction (for a recent review of connectivity methods, see Rossini et al., 2019). LLC was calculated using eLORETA (Pascual-Marqui, 2002; Pascual-Marqui et al., 2011); the eLORETA algorithm has been shown to accurately estimate the neural source of electrical signals detected by electrodes at the surface. We focused on regions of interest rather than electrode sites to improve our ability to localize the possible sources/regions that may be uniquely affected by distinct CR factors. Finally, LLC has been used to quantify the similarity of activity among brain regions in EEG studies of brain organization in healthy aging and in clinical decline (e.g., Vecchio et al., 2014, 2016), suggesting the sensitivity of the measure to capture differences in cortical connectivity in diverse populations.

We developed several hypotheses regarding the relationships between the different CR factors and brain connectivity. Overall, we predicted that participants with the highest CR levels would display higher local and long-range LLC values when compared to low-CR participants. Prior research has revealed greater connectivity over frontal regions and between frontal and posterior regions for delta and beta frequencies in participants with superior memory performance (Fleck et al., 2016), as well as greater theta power and connectivity in participants with higher cognitive function scores (Brickman et al., 2005; Douw et al., 2011). Therefore, we predicted that CR-related differences in LLC would be most substantial in delta, theta, and beta frequency bands.

We also generated specific predictions for the distinct CR factors (i.e., cognitive, social, and exercise). First, we anticipated that high local connectivity over frontal regions and high long-range connectivity would be present in participants with high cognitive CR. This prediction aligns with prior research that reported greater global coherence (Fleck et al., 2017) and higher global network efficiency (Li et al., 2009), in older adults with more years of formal education and higher IQs. For social CR, we predicted that greater LLC within the frontal lobes and between the frontal lobes and other cortical regions would be present in participants with high social CR levels, coinciding with prior research that has identified the importance of frontal lobe connectivity in social network organization (Cole et al., 2012). Finally, we predicted that exercise would enhance global network connectivity, resulting in greater long-range connectivity in participants with high physical engagement. This prediction was supported by prior research reporting increased brain volumes and greater functional connectivity in individuals with higher physical activity levels (Erikson et al., 2009; Arenaza-Urquijo et al., 2017; Sanchez-Lopez et al., 2018).

## Materials and Methods

### Participants

Data were collected from 108 adults, ages 35–75 years, living in and around Atlantic City, NJ, USA. To be eligible for the study, individuals needed to be right-handed, have no history of neurological disease or injury, no prior diagnosis of dementia or cognitive decline, have normal or corrected hearing and vision, and be able to read and write in English. Additionally, participants who were undergoing treatment for mood disorders, including anxiety and depression, were not eligible to participate.

During data collection, one participant met the “probable major depressive episode range” on *The Center for Epidemiologic Studies Depression-Revised* (Radloff, 1977) and the person’s associated data were excluded from analysis. In addition, two participants failed to complete the second research session and one participant had unusable EEG data, resulting in a final sample of 104. In our final sample, 76 participants were women (73.10%) and participants’ mean age was 56.59 years (*SD* = 7.55 years). Key demographics for these participants are presented in Table 1.

**Table 1:** Sample demographics.

		*n*	%	M	SD
Age				56.59	7.55
SES				46.95	9.48
Years of education				16.27	3.03
Depression total				6.23	5.58
Gender					
	Men	28	26.9		
	Women	76	73.1		
Race
	African American	25	24.0
	Asian/Pacific Islander	1	1.0
	Hispanic	9	8.7
	White	69	66.3
Employment status
	Employed	66	63.5
	Unemployed	10	9.6
	Retired	28	26.9
Annual income*
	Less than $25,000	10	10.0
	$25,000 to $49,000	31	31.0
	$50,000 to $74,999	21	21.0
	$75,000 to $99,000	22	22.0
	$100,000 to $250,000	16	16.0
Marital status
	Married	57	54.8
	Separated or divorced	24	23.1
	Never married	17	16.3
	Widowed	6	5.8
Education level
	High school graduate or GED	11	10.6
	Specialized training	4	3.8
	Some college	16	15.4
	Associate degrees	11	10.6
	Bachelor’s degree	32	30.8
	Some graduate or professional training	4	3.8
	Master’s degree	19	18.3
	Professional degree	5	4.8
	Doctoral degree	2	1.9

*Four participants declined to report income information.

### Materials

#### General Background Measures

##### Demographics Form

This form targeted important demographic variables, such as race/ethnicity, marital status, current living arrangements, country of origin, education, occupational attainment, literacy, computer use, hearing and vision, previous and current health conditions, neurological disorders, traumatic brain injury, concussion history, menopause status, drug and alcohol use, anxiety and depression, and physical activity level.

##### Edinburgh Handedness Inventory (EHI; Oldfield, 1971)

Handedness was assessed using a revised version of the Edinburgh Handedness Inventory (EHI), which included the 10 handedness items presented in Appendix II of Oldfield (1971). This version of the EHI has been used in numerous prior studies and is a reliable indicator of handedness (for a review of the measure, see Edlin et al., 2015). Participants indicate their hand preference (always left, usually left, no preference, usually right, or always right) for each item (e.g., writing, throwing a ball, opening a lid on a jar, etc.). Responses are awarded a point value: always left: −10, usually left: −5, no preference: 0, usually right: 5, and always right: 10, and the point values are summed across the 10 items to generate a handedness score. Participants with positive scores are considered to be right-handed.

##### Four Factor Index of Social Status (Hollingshead, 2011)

The index assesses four contributors to SES, which include education, occupation, marital status, and sex. Participants were asked to report their income, education, and most recent occupation. Education was scaled on a seven-point scale, from 1 (less than a seventh-grade education) to 7 (graduate degree). The occupation was scaled on a nine-step scale, from 1 (farm laborer/service worker) to 9 (higher executive/major professional). A composite SES variable was calculated by adding occupation score and education score (Education * 3) + (Income bracket * 5). If the participant was married, SES was calculated by finding the average score of the participant and their spouse.

##### The Center for Epidemiologic Studies Depression-Revised (CESD-R; Radloff, 1977)

The CESD-R is a 20-item screen for depression in the general population. The measure has shown to be a valid measure of depression for adults and older adults (Head et al., 2013). In addition to a total score, subscores are provided for depression, sadness, anhedonia, appetite, sleep, concentration, guilt, fatigue, agitation, and suicidal ideation.

#### Cognitive Reserve

##### General Ability Measure for Adults (GAMA; Naglieri and Bardos, 1997)

The general ability measure for adults (GAMA) is a brief, nonverbal IQ assessment for adults. The 66-item assessment is administered in 25 min and is appropriate for individuals from diverse language, cultural, and educational backgrounds. The GAMA provides scores on four subscales: matching, analogies, construction, and sequencing. In matching, participants must select the figure that is an exact match to the target item. In sequencing, participants select the item that completes the sequence to represent an abstract item’s path through space. For synthesis, participants must merge the presented items into a new composite figure. Finally, with analogy, participants must determine the relationship between two target items and apply that relationship to a new set of figures. The GAMA has high test-retest reliability and validity (Ispas et al., 2010), and performance on the GAMA correlates highly with other measures of IQ, such as the WAIS-R (Lassiter et al., 2000).

##### Social Network Index (SNI; Cohen et al., 1997)

The Social Network Index (SNI) measures the size and structure of social networks in adults and older adults in three areas: (a) network size: the number of people with whom the individual has regular contact; (b) network diversity: the number of different roles the individual plays in her/his social network (e.g., mother, church member); and (c) network embeddedness: the number of different social networks in which the individual is highly active.

##### Cognitive Reserve Index Questionnaire (CRIq; Nucci et al., 2012)

The Cognitive Reserve Index Questionnaire (CRIq) is a 24-item scale that captures CR-building activities beginning at age 18 years. The questionnaire targets engagement in cognitively stimulating activities across the lifespan and provides a total CRI score, along with subscores for CRI-education, CRI-occupation, and CRI-leisure.

##### Lifetime Physical Activity Questionnaire (Chasan-Taber et al., 2002)

The Lifetime Physical Activity Questionnaire includes 26 physical activities, such as lifting weights, bicycling, hiking, skiing, and housework. Participants were asked to estimate the number of hours per week, the number of months per year, and the number of years they completed each activity, from age 18 years to the present. A weighted formula was used to calculate the participant’s activity level in the past year (Exercise—Past Year), as well as their total lifetime physical activity level (Exercise—Lifetime). The distributions for the exercise variables were positively skewed and were log-transformed to increase normality.

#### Cognitive Function

##### Cambridge Neuropsychological Test Battery (CANTAB; Cambridge Cognition, Limited 2017)

We administered the Core Cognitive Battery from the CANTAB. This battery includes five measures and assesses cognitive differences in processing speed, sustained attention, episodic memory, and working memory. The measure is the most widely-used computerized assessment of cognition with older adults (Zygouris and Tsolaki, 2015) and has been shown to have high reliability and validity (Smith et al., 2013; Haring et al., 2015). The CANTAB is language and culture-independent and is sensitive to differences in cognition in normal aging vs. clinical decline (de Rover et al., 2011).

The *Motor Screening Test (MOT)* and the *Reaction Time Indicator (RTI)* are used to measure processing speed and motor speed. In the *MOT*, a cross appears in different locations on the screen and participants need to touch the location of the cross as quickly and accurately as possible. In the *RTI*, a yellow dot appears in one of five different circles. Participants are judged for their speed and accuracy in selecting the circle in which the dot appeared. Outcome measures include *RTI Simple—Median RT*, and *RTI Five Choice—Median RT*, with lower values indicating a faster processing speed.

*Rapid Visual Information Processing (RVP)* is a measure of sustained attention. During the task, numbers are rapidly presented (100 per minute) in the center of the computer screen and participants are instructed to press the screen when they detect the display of specific three-digit sequences (e.g., 3 4 6). Outcome measures include *RVP A Prime*, a measure of stimulus sensitivity, with higher scores indicating better sensitivity to target stimuli; and *RVP—Median RT*, with lower values indicating faster responses on correct trials.

The *Paired Associate Learning Test (PAL)* is a measure of episodic memory. In the *PAL*, boxes are displayed on the screen, with each box shielding a distinct pattern. The boxes are opened in random order, revealing the pattern behind the box. In the test phase, patterns are individually displayed in the center of the screen, and participants must press the box that shields the respective pattern. PAL outcome measures include *PAL Correct*, the number of trials in which the participant correctly recalled the entire sequence on trial 1, and *PAL Errors*, the number of errors made across trials.

Finally, the *Spatial Working Memory Test (SWM)* assesses spatial working memory. In the SWM test, boxes are presented on the computer screen and hidden behind one of the boxes is a yellow circle. Participants must find the box where the yellow circle is located. As the task progresses, the number of boxes on the screen increases. Performance is indicated by* SWM Errors*, the number of times participants revisit a box where the yellow circle has already been found and *SWM Strategies*, the number of times participants begin a new search strategy from the same box. Low scores on the SWM Strategies variable indicate more restarts from the same location, reflecting the application of a planned strategy when searching for the yellow circle. Thus, low scores on SWM Errors and low scores on SWM Strategies indicate the best SWM performance.

#### Electroencephalography (EEG)

EEG data were recorded using a 129-channel HydroCel Geodesic Sensor Net, with Cz reference (Electrical Geodesics, Inc.). Sensor impedance levels were below 50 KΩ, appropriate for use with the Net Amps 400 high-impedance amplifier. Data were sampled at 250 Hz and filtered using an analog 0.1–100 Hz band-pass filter. All data were recorded using Net Station 5.2 software. Recordings included 3 min of resting-state data with eyes open followed by 3 min of resting-state data with eyes closed.

**Figure 1:**
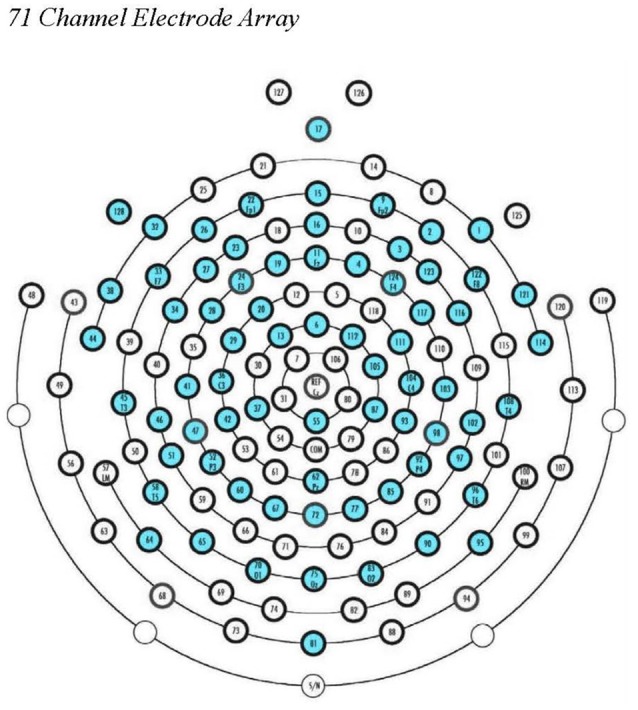
EGI 128-channel Hydrocel Geodesic Sensor Net. The 71 source electrode locations used in our ROI calculations in eLORETA are noted in blue.

EEG data were processed offline using EEGLAB 14 (Delorme and Makeig, 2004), supplemented by MATLAB scripts, run using Matlab 2017a (Mathworks, Natick, MA, USA). Data were filtered in EEGLAB using a band-pass filter (1–47 Hz), downsampled to 71 electrodes (see Figure 1), and segmented into 2-s epochs. After visual inspection to remove gross artifacts and bad channels, we used the AMICA plugin for EEGLab to perform ICA decomposition. The resulting components were reviewed by a trained researcher and artifactual components were removed. We then interpolated missing channels (mean number of interpolated channels = 1.14; *SD* = 1.84), re-referenced to average reference, and exported the cleaned data files for signal processing and analysis in LORETA (Fuchs et al., 2002; Pascual-Marqui, 2002; Jurcak et al., 2007). For a participant’s EEG data to be retained for analysis, a minimum of 2 min of clean EEG data was required in the eyes-open block and 2 min of clean EEG data in the eyes-closed block. The mean number of artifact-free epochs that remained per participant across the eyes-open and eyes-closed blocks was 158.34 (*SD* = 14.02; an average of 5.28 min of clean data per participant).

**Figure 2:**
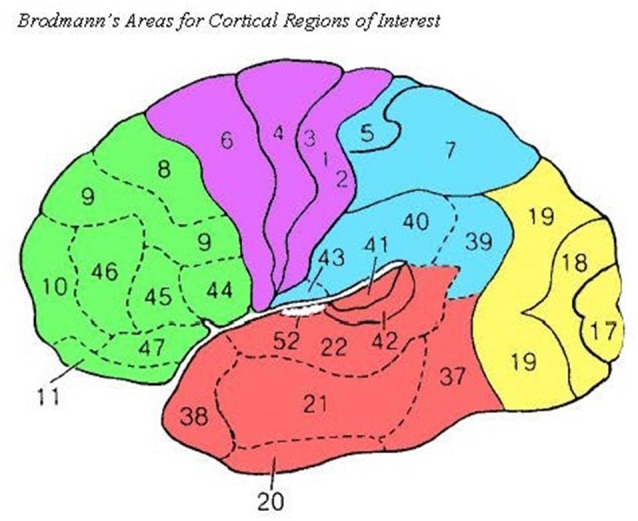
Source Brodmann’s Areas comprising cortical ROIs are noted above as in Babiloni et al. (2017). Frontal (green): 8, 9, 10, 11, 44, 45, 46, and 47; Central (purple): 1, 2, 3, 4, and 6; Temporal (red): 20, 21, 22, 37, 38, 41, and 42; Parietal (blue): 5, 7, 30, 39, 40, and 43; Occipital (yellow): 17, 18, and 19.

Within the LORETA software program, we used the eLORETA algorithm for inverse source reconstruction. Using LORETA, we calculated the LLC (Pascual-Marqui et al., 2011) among 58 cortical ROIs using the *All Nearest Voxels Method*; ROIs corresponded to 29 Brodmann’s areas in the left and right hemispheres that mapped onto five cortical regions, as was done in Babiloni et al. (2017; see Figure 2). LLC quantifies the linear relatedness of the signals in the frequency domain between ROIs, excluding zero phase connectivity, which may be heavily influenced by volume conduction. As a result, the LLC measure reflects the electrical similarity between brain regions (for a review, see Pascual-Marqui et al., 2011). LLC measures were calculated for the following frequency bands: delta (1.0–4.0 Hz), theta (4.0–8.0 Hz), low alpha (8.0–10.5 Hz), high alpha (10.5–13.0 Hz), low beta (13.0–20.0 Hz), high beta (20.0–30.0 Hz), and gamma (30.0–45.0 Hz).

### Procedure

The protocol for this research was approved by Stockton University’s Institutional Review Board. All participants provided written informed consent. Participants were tested in two 1.5-h sessions, scheduled approximately 1 week apart (*M* intersession interval = 8.43 days; *SD* = 3.36).

In session 1, participants completed the handedness form, CESD-R, and the demographics form, which contained questions that were necessary to calculate socioeconomic status. After the demographics form, participants completed the GAMA IQ assessment or the EEG recording, the order of which was counterbalanced across participants. In the EEG recording, the EEG net was applied, and participants had their resting-state brain activity recorded for 3 min with eyes open followed by 3 min with eyes closed. Participants were relaxed, but awake and were instructed to keep their minds free from wandering thoughts. Participants’ EEG recordings were monitored for drowsiness by a trained research assistant, and a verbal prompt was issued in the event any drowsiness was detected. For the GAMA, participants worked through four practice problems under the supervision of the Experimenter, before completing the assessment. Participants were given 25 min to complete as many problems on the GAMA as possible. At the end of session 1, participants were compensated $25 and were scheduled for Session 2.

In Session 2, participants completed the CANTAB along with several self-report measures of lifestyle to estimate CR; the administration of the CANTAB and CR measures was counterbalanced. Within the CANTAB, all participants completed the five assessments in the same order: (a) MOT; (b) RTI; (c) PAL; (d) RVP; and (e) SWM. The CR measures in session 2 included the SNI, the CRIq, and the Lifetime Physical Activity Index. Presentation of the CR measures was counterbalanced. After the session, participants were thanked for their participation and were compensated $50.

### EEG Data Reduction

To explore cortical connectivity, we calculated connectivity within cortical regions (i.e., local connectivity) and between cortical regions within the same hemisphere (i.e., long-range connectivity). Local connectivity was calculated within frontal, central, temporal, parietal, and occipital regions, separately for each hemisphere, by calculating the mean LLC for all combinations of ROI pairs within that cortical region (e.g., left frontal LLC, was the mean of LLC values between all combinations of ROIs that corresponded to frontal Brodmann’s areas in the left hemisphere: BAs 8, 9, 10, 11, 44, 45, 46, and 47). Long-range connectivity was calculated for each cortical region, separately for each hemisphere, by calculating the mean LLC between that region of the cortex and each of other cortical regions in that hemisphere (e.g., left frontal was calculated as the mean LLC for LF and left central, LF and left temporal, LF and left posterior, and LF and left occipital. In the end, for each frequency band, we had 10 LLC measures of local connectivity and 10 LLC measures of long-range connectivity.

All LLC variables were subject to square root transformations to increase the normality of the distributions. After the square root transformations, LLC variables were explored for the presence of univariate outliers, and any outliers (*z* > 3.29) were replaced with a value that was 0.01 more extreme than the next most extreme value in the dataset (Tabachnick and Fidell, 2013). Finally, Mahalanobis distance (*p* < 0.001) was used to test for the presence of multivariate outliers separately for local and long-range variables. No multivariate outliers were detected.

## Results

### Statistical Analyses

We began by forming high-CR and low-CR groups. Because diverse lifestyle factors may have distinct contributions to the brain connectivity-cognition relationship, we explored differences between CR groups generated using CR factor scores. To detect separable CR factors, we conducted principal factors extraction on the six CR variables using Promax rotation. Using a Scree plot and Eigenvalues greater than 1.0, three factors were extracted. These factors explained 69.90% of the total variance in the data. We used a factor loading cutoff of 0.45 to include a variable in the interpretation of the factor. Years of education, GAMA IQ, and CRI-Occupation loaded on factor 1 (CR-COGNITIVE); CRI-Leisure and Social Network loaded on factor 2 (CR-SOCIAL), and Exercise—Past Year and Gama IQ (negative) loaded on factor 3 (CR-EXERCISE). To explore differences in connectivity associated with each factor, we created high-CR and low-CR groups for CR-COGNITIVE, CR-SOCIAL, and CR-EXERCISE subcomponents using a median split on the relevant factor score. There were no missing data for the CR variables. However, for the cognitive measures, missing data were addressed by substituting the sample mean on the variable for the missing score.

We conducted mixed-model ANOVAs to test connectivity differences in LLC values between high- and low-CR groups. Separate analyses were conducted for local connectivity and long-range connectivity for each frequency band, under eyes-open and eyes-closed recording conditions. We conducted 2 × 2 × 2 × 2 × 5 ANOVAs with CR-COGNITIVE group, CR-SOCIAL group, and CR-EXERCISE group as between-subjects variables, and hemisphere (left, right), and region (frontal, central, temporal, parietal, occipital) as within-subjects variables. Age and gender were used as covariates in all analyses. All results are reported as two-tailed analyses and repeated-measures ANOVAs are reported as Huynh-Feldt corrected.

We limit our discussion of findings to significant main effects or interactions involving at least one CR-grouping variable as a factor. Because we conducted separate comparisons for local and long-range LLC for each frequency band, we used a corrected alpha level of *p* < 0.001, to identify significant main effects or interactions.

### Local LLC

We began by conducting mixed-model ANOVAs to assess CR-group differences in local connectivity. These analyses were conducted to test our hypothesis that higher CR levels, particularly in cognitive and social domains, would be associated with greater local connectivity. For participants high in social CR, we specifically predicted that this effect would be most evident over frontal regions. We also predicted that these effects would be strongest in delta, theta, and beta frequency bands.

#### CR-COGNITIVE

**Figure 3:**
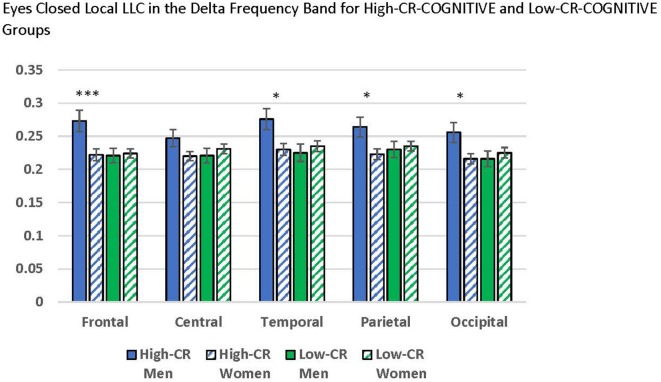
Comparisons between men and women who were members of the high-cognitive reserve (CR) COGNITIVE group revealed higher local lagged linear connectivity (LLC) values for men than women in the eyes-closed delta band for frontal, temporal, parietal, and occipital regions. No differences were found between men and women who were members of the low-CR-COGNITIVE group. LLC values are reported as square root transformed and error bars reflect one standard error of the mean. *p < 0.05, **p < 0.01, ***p < 0.001.

Our ANOVAs revealed a significant CR-COGNITIVE × Region interaction for the delta frequency band under eyes-closed recording conditions, *F*_(4,376)_ = 5.601, *p* < 0.001, $\eta_{\text{p}}^{2}$ = 0.056. *Post hoc* univariate ANOVAs comparing CR-COGNITIVE groups for each region were not significant (*p* > 0.05). However, *post hoc* repeated-measures ANOVAs exploring differences in local LLC values among regions, separately for high-CR-COGNITIVE and low-CR-COGNITIVE groups revealed a significant Gender × Region interaction within the high-CR-COGNITIVE group that was not present in the low-CR-COGNITIVE group (high CR: *F*_(4,196)_ = 3.834, *p* = 0.005, $\eta_{\text{p}}^{2}$ = 0.073; low CR: *F*_(4,196)_ = 0.419, *p* = 0.795, $\eta_{\text{p}}^{2}$ = 0.004). *Post hoc* univariate ANOVAs between women and men in the high-CR-COGNITIVE group revealed greater local LLC values in high-CR men than high-CR women for frontal, temporal, parietal, and occipital regions (frontal: *F*_(1,49)_ = 7.580, *p* = 0.008, $\eta_{\text{p}}^{2}$ = 0.134; temporal: *F*_(1,49)_ = 6.069, *p* = 0.017, $\eta_{\text{p}}^{2}$ = 0.110; parietal: *F*_(1,49)_ = 5.451, *p* = 0.024, $\eta_{\text{p}}^{2}$ = 0.100; occipital: *F*_(1,49)_ = 5.303, *p* = 0.026, $\eta_{\text{p}}^{2}$ = 0.098). The result for the comparison for the central region was in the same direction but failed to achieve significance (*F*_(1,49)_ = 2.987, *p* = 0.090, $\eta_{\text{p}}^{2}$ = 0.057; see Figure 3).

No significant local LLC differences were identified in our eyes-open data or in the other eyes-closed frequency bands.

#### CR-SOCIAL

**Figure 4:**
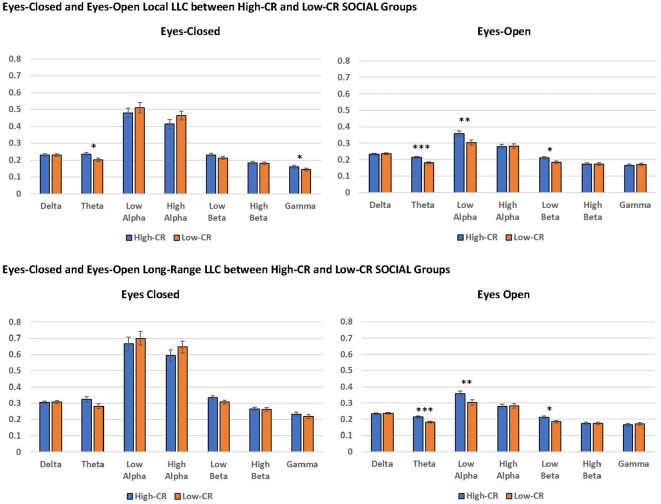
The high-CR-SOCIAL group exhibited greater eyes-open local LLC values in theta (*p* < 0.001) and low alpha (*p* = 0.005), than the low-CR-SOCIAL group. Additionally, the high-CR-SOCIAL group exhibited greater long-range LLC values for theta (*p* < 0.001) and low alpha (*p* = 0.006) than the low-CR-SOCIAL group. LLC values are reported as square root transformed and error bars reflect one standard error of the mean. *p < 0.05, **p < 0.01, ***p < 0.001.

Mixed-model ANOVAs revealed significant main effects and interactions for CR-SOCIAL in our eyes-open data. We observed a significant CR-SOCIAL main effect in the theta frequency band for eyes-open local LLC values, *F*_(1,94)_ = 16.060 *p* < 0.001, $\eta_{\text{p}}^{2}$ = 0.146, along with a marginally significant CR-SOCIAL main effect in low alpha, *F*_(1,94)_ = 8.385, *p* = 0.005, $\eta_{\text{p}}^{2}$ = 0.082. For eyes-open theta and low alpha, high-CR-SOCIAL participants displayed higher overall local LLC values throughout the brain than low-CR-SOCIAL participants (Theta: Low-CR: *M* = 0.181, *SEM* = 0.006; High-CR: *M* = 0.213, *SEM* = 0.006; Low Alpha: Low CR: *M* = 0.303, *SEM* = 0.016; High CR: *M* = 0.359, *SEM* = 0.016). Mean local LLC values are displayed for high-CR and low-CR-SOCIAL groups for each frequency band in Figure 4.

**Figure 5:**
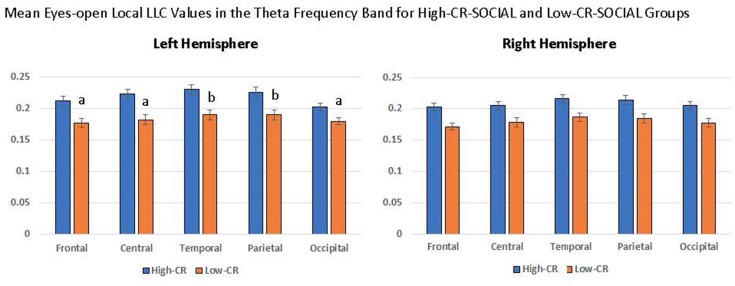
High-CR-SOCIAL participants consistently exhibited higher eyes-open local LLC values than low-CR-SOCIAL for the theta frequency band. The distribution of LLC values within CR groups differed in the left hemisphere for low-CR-SOCIAL but not high-CR-SOCIAL participants. Low-CR-SOCIAL participants displayed higher local LLC values over the temporal and parietal regions when compared to the frontal region (*p* < 0.001), central region (*p* < 0.01), and occipital region (*p* < 0.001). Local LLC values in the left temporal and parietal regions did not differ from each other (*p* > 0.05). Error bars indicate one standard error of the mean and all LLC values are reported as square-root transformed. All ‘a’ conditions differed significantly from ‘b’ conditions but did not differ significantly from other ‘a’ conditions. Similarly, ‘b’ conditions differed significantly from ‘a’ conditions but not from each other.

In addition, our analyses revealed a significant CR-SOCIAL × Hemisphere × Region interaction in eyes-open theta, *F*_(4,376)_ = 5.651, *p* < 0.001, $\eta_{\text{p}}^{2}$ = 0.057. *Post hoc* mixed-model ANOVAs conducted separately for the left and right hemispheres, using CR-group and Region as variables, revealed a significant CR-SOCIAL × Region interaction for the left hemisphere, *F*_(4,400)_ = 3.776, *p* = 0.007, $\eta_{\text{p}}^{2}$ = 0.036, but not the right hemisphere, *F*_(4,400)_ = 0.419, *p* = 0.750, $\eta_{\text{p}}^{2}$ = 0.004. Local LLC values in eyes-open theta were higher in high-CR-SOCIAL than low-CR-SOCIAL participants for all regions in the left hemisphere (Frontal: *F*_(1,100)_ = 12.869, *p* < 0.001, $\eta_{\text{p}}^{2}$ = 0.114; Central: *F*_(1,100)_ = 12.723, *p* < 0.001, $\eta_{\text{p}}^{2}$ = 0.113; Temporal: *F*_(1,100)_ = 12.713, *p* < 0.001, $\eta_{\text{p}}^{2}$ = 0.113; Parietal: *F*_(1,100)_ = 10.853, *p* = 0.001, $\eta_{\text{p}}^{2}$ = 0.098; Occipital: *F*_(1,100)_ = 7.466, *p* = 0.007, $\eta_{\text{p}}^{2}$ = 0.069). *Post hoc* repeated-measures ANOVAs testing differences among left-hemisphere regions, separately for high-CR-SOCIAL and low-CR-SOCIAL groups, revealed a significant difference in local LLC values among regions for the low-CR group but not the high-CR group (Low-CR: *F*_(4,196)_ = 2.577, *p* = 0.048, $\eta_{\text{p}}^{2}$ = 0.050; High-CR: *F*_(4,196)_ = 1.285, *p* = 0.277, $\eta_{\text{p}}^{2}$ = 0.026. *Post hoc* LSD comparisons revealed greater local LLC values in temporal and parietal regions, when compared to frontal (*p* < 0.001), central (*p* < 0.01), and occipital regions (*p* < 0.001). Temporal and parietal regions did not differ significantly from each other (*p* > 0.05; see Figure 5).

**Figure 6:**
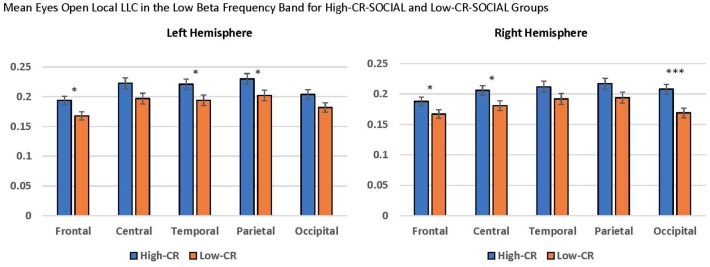
Local LLC values were higher in the high-CR-SOCIAL group than the low-CR-SOCIAL group over the right occipital region (*p* = 0.001). Error bars reflect the standard error of the mean and connectivity values are reported as square-root transformed. *p < 0.05, **p < 0.01, ***p < 0.001.

Finally, we observed a significant CR-SOCIAL × Hemisphere × Region interaction in low beta for eyes-open local LLC values, *F*_(4,376)_ = 5.399, *p* < 0.001, $\eta_{\text{p}}^{2}$ = 0.054. *Post hoc* mixed-model ANOVAs, conducted separately for the left and right hemispheres revealed a significant CR-SOCIAL × Region interaction in the right hemisphere, *F*_(4,400)_ = 4.292, *p* = 0.004, $\eta_{\text{p}}^{2}$ = 0.041, but not the left hemisphere, *F*_(4,400)_ = 0.327, *p* = 0.827, $\eta_{\text{p}}^{2}$ = 0.003. Again, local LLC values were greater in high-CR-SOCIAL participants than low-CR-SOCIAL participants for frontal, central and occipital regions (Frontal: *F*_(1,100)_ = 4.268, *p* = 0.041, $\eta_{\text{p}}^{2}$ = 0.041; Central: *F*_(1,100)_ = 4.708, *p* = 0.032, $\eta_{\text{p}}^{2}$ = 0.045; Occipital; *F*_(1,100)_ = 11.334, *p* = 0.001, $\eta_{\text{p}}^{2}$ = 0.102), but failed to achieve significance for temporal or parietal regions (*p* > 0.05; see Figure 6). *Post hoc* repeated-measures ANOVAs exploring differences in local LLC among regions in the right hemisphere, separately for high-CR-SOCIAL and low-CR-SOCIAL groups, were not significant (*p* > 0.05).

No significant local LLC differences were identified for CR-Social groups in eyes-closed recordings or for the other frequency bands in our eyes-open data.

#### CR-EXERCISE

Although we made no specific predictions for local connectivity in high-CR vs. low-CR-EXERCISE groups, we included the CR-EXERCISE variable as a grouping variable in our mixed-model ANOVAs for local LLC. No significant main effects or interactions were detected in our eyes-closed or eyes-open data for the CR-EXERCISE factor.

#### Long-Range LLC

Next, we conducted mixed-model ANOVAs to assess CR-group differences in long-range connectivity. These analyses were conducted to test our hypothesis that higher CR levels, particularly in the cognitive and exercise domains, would be associated with greater long-range connectivity. We also predicted that these effects would be strongest in delta, theta, and beta frequency bands.

#### CR-COGNITIVE

We observed a marginally significant CR-COGNITIVE × Region interaction in eyes-closed low alpha, *F*_(4,376)_ = 4.453, *p* = 0.003, $\eta_{\text{p}}^{2}$ = 0.043. *Post hoc* univariate ANOVAs were used to compare the CR groups for each of the five brain regions. Comparisons between high-CR and low-CR-COGNITIVE groups revealed greater long-range LLC values for the occipital region in high-CR than low-CR participants, *F*_(1,94)_ = 4.575, *p* = 0.035, $\eta_{\text{p}}^{2}$ = 0.044 (High-CR: *M* = 0.884, *SEM* = 0.054; Low-CR: *M* = 0.720, *SEM* = 0.054), suggesting greater connectivity between the occipital lobes and other regions of the brain in low alpha. No other significant differences were observed between regions (*p* > 0.05). Additionally, no significant main effects or interactions were observed for long-range LLC in our eyes-open data or for other frequency bands in our eyes-closed data.

#### CR-SOCIAL

For high-CR and low-CR-SOCIAL groups, we detected a significant main effect for long-range LLC for eyes open theta, *F*_(1,94)_ = 10.857, *p* < 0.001, $\eta_{\text{p}}^{2}$ = 0.104, as well as a marginally significant main effect for CR-SOCIAL in low alpha, *F*_(1,94)_ = 4.268, *p* = 0.041, $\eta_{\text{p}}^{2}$ = 0.041 (see Figure 4). In both instances, the high-CR SOCIAL group exhibited greater eyes-open LLC than the low-CR SOCIAL group (Theta: High-CR: *M* = 0.310, *SEM* = 0.011; Low-CR: *M* = 0.261, *SEM* = 0.010; Low Alpha: High-CR: *M* = 0.513, *SEM* = 0.024; Low-CR: *M* = 0.418, *SEM* = 0.023. No significant differences were observed for CR-SOCIAL groups in our eyes-closed data or in the other eyes-open frequency bands.

#### CR-EXERCISE

Finally, our analysis of long-distance LLC revealed a marginally significant CR-EXERCISE × Hemisphere interaction in eyes-closed theta, *F*_(1,94)_ = 7.815, *p* = 0.006, $\eta_{\text{p}}^{2}$ = 0.077. *Post hoc* univariate ANOVAs comparing High-CR and Low-CR-EXERCISE groups failed to find a significant difference between CR-groups for the left hemisphere, *F*_(1,94)_ = 2.431, *p* = 0.122, $\eta_{\text{p}}^{2}$ = 0.025, or the right hemisphere, *F*_(1,94)_ = 0.010, *p* = 0.919, $\eta_{\text{p}}^{2}$ < 0.001. The trend was for low-CR participants to exhibit greater long-distance LLC in the left hemisphere than high-CR participants (LEFT: High-CR: *M* = 0.301, *SEM* = 0.017; Low-CR: *M* = 0.341, *SEM* = 0.018; RIGHT: High-CR: *M* = 0.297, *SEM* = 0.017; Low-CR: *M* = 0.300, *SEM* = 0.017). No significant differences were observed for CR-EXERCISE groups in our eyes-open data or in the other eyes-closed frequency bands.

### Covariates

#### Gender

**Figure 7:**
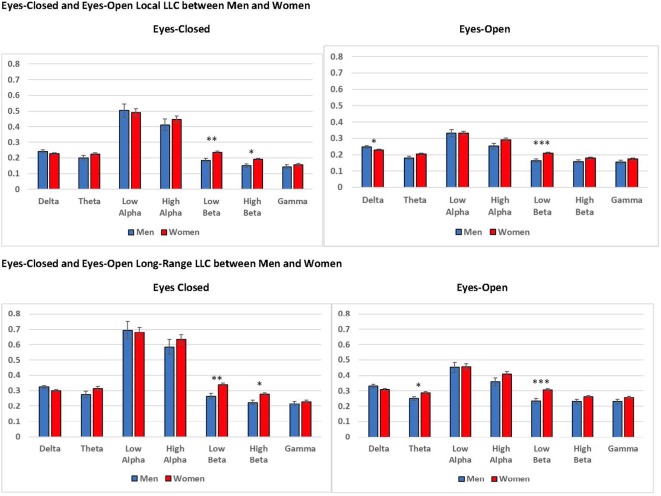
Differences in eyes-closed and eyes-open local LLC values and long-range LLC values between men and women. Women exhibited higher local LLC values in low beta for eyes-open and eyes-closed recording conditions than men (open: *p* < 0.001, closed *p* = 0.002). Similarly, women presented higher long-range LLC values in low beta for eyes-open and eyes-closed recording conditions (open: *p* < 0.001, closed *p* = 0.002). All LLC values are presented as square root transformed and error bars indicate one standard error of the mean. *p < 0.05, **p < 0.01, ***p < 0.001.

We observed several significant findings involving the gender covariate. For local LLC, we observed a significant gender main effect for eyes-open low beta, *F*_(1,94)_ = 12.318, *p* < 0.001, $\eta_{\text{p}}^{2}$ = 0.116, and a marginally significant gender main effect for eyes-closed low beta, *F*_(1,94)_ = 10.619, *p* = 0.002, $\eta_{\text{p}}^{2}$ = 0.102. In both cases women exhibited higher local LLC values than men (eyes-open: men: *M* = 0.163, *SEM* = 0.011; women: *M* = 0.210, *SEM* = 0.006; eyes-closed: men: *M* = 0.184, *SEM* = 0.012; women: *M* = 0.236, *SEM* = 0.007; see Figure 7). Additionally, we observed marginally significant gender × region interactions for eyes-closed theta, *F*_(4,376)_ = 4.579, *p* = 0.002, $\eta_{\text{p}}^{2}$ = 0.046, and low alpha, *F*_(4,376)_ = 4.006, *p* = 0.007, $\eta_{\text{p}}^{2}$ = 0.041. Although the trend was for women to exhibit higher local LLC values than men, none of the *post hoc* univariate ANOVAs comparing differences between men and women by region for these frequency bands was significant (*p* > 0.05).

In addition to our local LLC findings, we observed a significant gender main effect for long-range LLC in eyes-open low beta, *F*_(1,94)_ = 12.782, *p* < 0.001, $\eta_{\text{p}}^{2}$ = 0.120, along with a marginally significant main effect for gender for eyes-closed low beta, *F*_(1,94)_ = 10.184, *p* = 0.002, $\eta_{\text{p}}^{2}$ = 0.098. Again, in both cases, women displayed higher long-range LLC values than men (eyes-open: men: *M* = 0.232, *SEM* = 0.016; women: *M* = 0.305, *SEM* = 0.009; eyes-closed: men: *M* = 0.265, *SEM* = 0.018; women: *M* = 0.341, *SEM* = 0.011; see Figure 7).

#### Age

Age was not a significant covariate in any of our analyses.

### LLC and Cognitive Function

**Figure 8:**
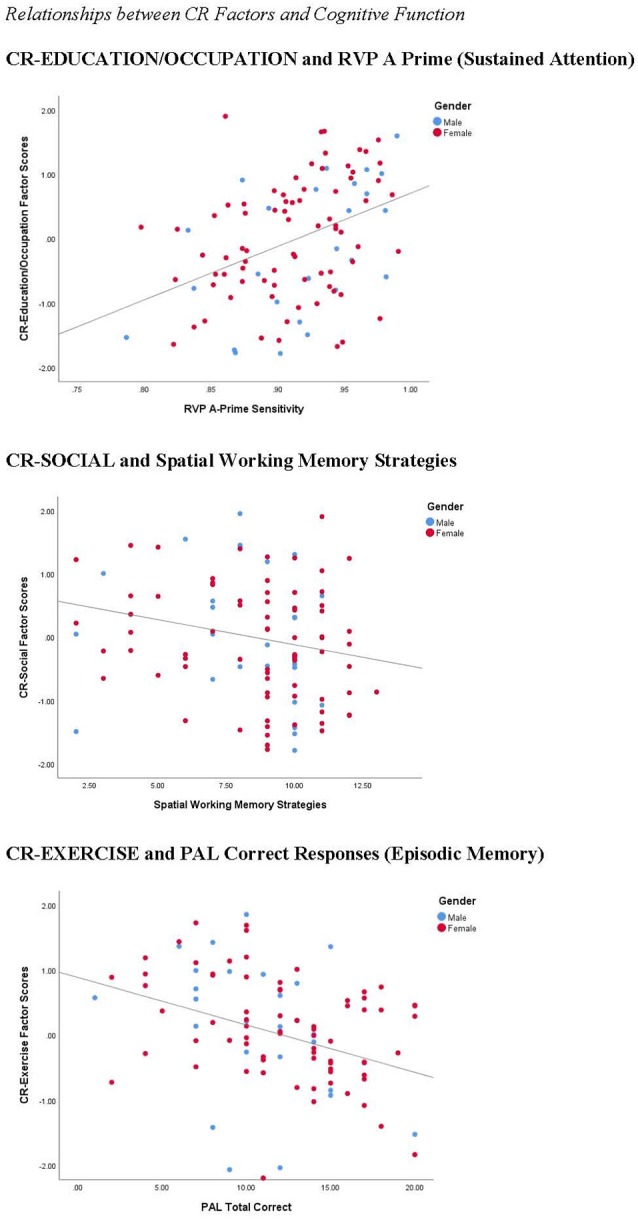
Correlations between CR factors and cognitive function. CR-COGNITIVE scores were positively correlated with performance on the rapid visual information processing (RVP; *r* = 0.392, *p* < 0.001), a measure of sustained attention. CR-SOCIAL scores were inversely correlated with Spatial Working Memory Strategies (*r* = −0.330, *p* < 0.001), indicating stronger working memory performance in participants with high scores on this factor. Finally, surprisingly, CR-Exercise factor scores were inversely correlated with the number of correct responses on the PAL (*r* = −0.251, *p* < 0.05), a measure of episodic memory, suggesting weaker memory performance in participants with the highest scores on this factor.

We explored the relationship between our measures of cognitive function and the three CR factors (see Table 2). For the CR variables, we conducted partial correlations to explore the relationship between CR factor scores and cognitive function measures, controlling for age. For the separate CR factors, CR-COGNITION factor scores were significantly correlated with SWM and RVP A-Prime scores, with the strongest relationship between CR-COGNITION and RVP A-Prime (see Figure 8). CR-SOCIAL was significantly correlated with the SWM measures, with the strongest relationship present for SWM Strategies (see Figure 8). Finally, CR-EXERCISE was significantly correlated with the PAL measures, and the RVP Median RT measure. Although the strongest relationships were present between CR-EXERCISE and PAL scores, the correlations were such that higher scores on the CR-EXERCISE factor were associated with fewer correct responses and more errors on the PAL (see Figure 8).

**Table 2:** Relationships between cognitive function and cognitive reserve (CR).

	CR-COGNITIVE	CR-SOCIAL	CR-EXERCISE
PAL Correct	0.139	0.198	−0.251*
PAL Errors	−0.159	−0.181	0.226*
RTI Five-choice Median RT	−0.051	0.029	−0.022
RTI Simple Median RT	−0.043	0.126	0.034
RVP A Prime	0.392***	0.076	−0.186
RVP Median RT	−0.132	−0.098	0.210*
SWM Errors	−0.222*	−0.297**	0.017
SWM Strategies	−0.203*	−0.330***	−0.065

*p < 0.05, **p < 0.01, ***p < 0.001.

Although differences in LLC values, along with differences in cognitive function were present between high and low CR-groups, for cognitive and social groupings, we failed to find significant relationships directly between the connectivity measures that distinguished groups (e.g., for CR-SOCIAL, eyes open theta) and measures of cognitive function.

## Discussion

We conducted the present research to examine the influence of cognitive, social, and physical CR factors on functional brain connectivity and cognitive function in healthy adults and older adults. Cortical connectivity was calculated for regions of interest using LLC (Pascual-Marqui, 2002; Pascual-Marqui et al., 2011). LLC reflects the similarity of electrical activity among brain regions, excluding linear connectivity with zero phase, which could be affected by volume conduction. We explored differences between cognitive, social, and physical CR-groups in local and long-range connectivity in participants’ resting-state EEG recordings.

Overall, we predicted that participants high in CR would exhibiter higher local and long-range LLC values when compared to participants low in CR and that these effects would be most evident in delta, theta, and beta frequency bands. For local connectivity, the high-CR-COGNITIVE group demonstrated higher local LLC than the low-CR-COGNITIVE group in the eyes-closed delta, though this effect was specific to male participants. Additionally, the high-CR-SOCIAL group exhibited greater local LCC than the low-CR-SOCIAL group in theta and low alpha, under eyes-closed and eyes-open recording conditions. For long-range connectivity, high-CR-COGNITIVE participants displayed greater eyes-closed connectivity than low-CR-COGNITIVE participants between the occipital lobe and other cortical regions for low alpha. Similar to local LLC, the high-CR-SOCIAL group demonstrated greater long-range LLC in both eyes-closed and eyes-open recording conditions for theta and low alpha. Finally, there was a trend for the low-CR-EXERCISE group to demonstrate higher long-range connectivity among regions in the left hemisphere, when compared to the low-CR-EXERCISE group; this was the only instance when low-CR scores on any factor were associated with higher connectivity values. We interpret these findings below.

For the CR-COGNITIVE factor, participants who were members of the high-CR group exhibited greater eyes-closed LLC between the occipital lobes and other cortical regions in low alpha, supporting our prediction that greater long-range connectivity would be present in high-CR than low-CR-individuals. Furthermore, high cognitive factor scores were associated with stronger performance on measures of spatial working memory and sustained attention. These findings align with prior research from our lab reporting positive correlations between global coherence and CR (Fleck et al., 2017). In our prior work, older sample members who were high in CR, estimated using education and IQ, exhibited higher eyes-closed coherence in low alpha and high alpha frequencies, when compared to older participants low in CR. Additionally, high-CR participants displayed higher scores on measures of executive function and memory than low-CR participants (Fleck et al., 2017). Moreover, other researchers have consistently shown that higher task-related alpha coherence is associated with better working memory performance in healthy adults (Hogan et al., 2003; Sauseng et al., 2005, 2010). Thus, we suggest that greater long-range connectivity in our participants with high cognitive factor scores may signify a neural mechanism that supports superior cognition in older adults with high CR.

As predicted, high-CR-COGNITIVE group members also displayed greater local connectivity when compared to low-CR-COGNITIVE group members. In this case, men in the high-CR group displayed greater eyes-closed local connectivity in the delta frequency band. Although our hypotheses stated that delta would be one of the frequency bands to reflect CR-related effects, it is not clear why this effect was present only in men. While the gender covariate was not statistically significant in eyes-closed delta using our modified alpha level, we did detect a trend for greater local connectivity in delta for men than women (see Figure 7, eyes closed: *p* = 0.111; eyes-open: *p* = 0.027). This trend aligns with prior research conducted by Douw et al. (2011) who observed greater local and long-range connectivity in men than women, specifically for delta. Considering research reporting differences in aging between men’s and women’s brains (e.g., Király et al., 2016), delta connectivity may reflect a unique protective mechanism in men.

In line with our hypotheses, we found several differences in local LLC between participants who were high vs. low in CR social factor scores, confirming the importance of social engagement in building CR (e.g., Yates et al., 2016). However, in our research, participants who were more socially engaged exhibited greater local connectivity and increased long-range connectivity in theta and low alpha when compared to participants who were less socially engaged. Our findings for the CR-SOCIAL factor support the importance of social engagement in cognitive function that has been reported in prior research. Evans et al. (2018) observed a decline in cognitive function in community members who had few ties to family or friends, whereas engaging in social and cognitive leisure activities in middle (Ihle et al., 2018) and older age (Fancourt and Steptoe, 2018) produced positive outcomes for cognition in later life. Furthermore, some research has even suggested that social engagement may be more important than cognitive function in reducing the degree of cognitive decline that occurs during aging (Miceli et al., 2019).

We suspect that the influence of social engagement specifically on theta and low alpha frequencies is an important component in explaining the contributions of social engagement to long-term cognitive function. Numerous prior studies have reported relationships between theta and low alpha power and coherence and cognitive function in older adults (e.g., Klimesch, 1999; Solomon et al., 2017). Furthermore, theta and low alpha frequencies are significantly affected during aging. Reductions in task-related theta power over the frontal midline region has been reported in aging (Kardos et al., 2014). Moreover, researchers have shown that age is inversely related to alpha power (for a review, see Klimesch, 1999), particularly over posterior regions, with a stronger relationship between alpha power and cognition in older adults experiencing a clinical decline. The presence of high LLC in our participants who were high in social engagement may reflect the positive outcomes of a socially-stimulating lifestyle, offering protection against age-related cognitive decline.

Contrary to our hypothesis, we failed to find greater long-range LLC in participants with high CR-EXERCISE factor scores. Although prior research has reported improved cognitive function and/or increased cortical power with increased physical exercise (Sanchez-Lopez et al., 2018; Reas et al., 2019), we did not observe significant differences in LLC between exercise groups. We did detect a trend toward a significant CR-EXERCISE × Hemisphere interaction for long-range connectivity in eyes-closed theta, but the low-CR group trended toward higher LLC values over the left hemisphere than the high-CR group. Moreover, in our sample, higher CR exercise factor scores were associated with weaker performance on the PAL measure of episodic memory and longer reaction times during the RVP sustained attention test. However, we speculate that this pattern may be a byproduct of the variables that loaded on the exercise factor in our factor analysis. In the present research, exercise level in the past year loaded positively on the exercise factor, while IQ scores loaded negatively, such that a low IQ score coupled with high physical engagement in the past year was associated with the highest exercise factor scores. However, when we controlled for lifetime physical activity in our correlation analyses, high exercise factor scores were associated with faster processing speeds, more closely aligning with the beneficial effects of exercise observed in prior research (*r* = −0.229, *p* = 0.021).

In addition to the CR-related effects that were the focus of the present research, we observed differences in connectivity between men and women. In our findings, gender effects were present in low beta for local and long-range connectivity, under eyes-closed and eyes-open recording conditions, with women displaying higher LLC values than men for all measures. Prior research has reported that women’s brains age more slowly than men’s, offering increased protection against decline (Király et al., 2016; Goyal et al., 2019). Greater connectivity in the low beta frequency band in women than men may reflect healthier brain aging and the potential for sustained cognition in women. Prior research in our lab observed a positive association between beta coherence and cognitive function in older adults experiencing healthy aging (Fleck et al., 2016). In our prior work, increased coherence between frontal and posterior brain regions in the beta frequency band was exhibited by participants with the strongest working memory and long-term memory performance. The strength and consistency of the differences in low beta connectivity between men and women suggest its importance for further exploration in future research.

We note several limitations that should be considered when weighing the potential impact of our research. First, our sample was limited to healthy adults and older adults who volunteered to participate in our research, with no prior history of cognitive decline. As a result, we likely truncated the range of CR levels, as well as cognitive abilities, in our sample minimizing differences between CR groups. Further, we used the CANTAB Core Cognitive Battery to quantify cognitive function in participants. This measure is a computerized, nonverbal assessment of cognition, which may bias performance on the measure toward individuals with significant computer experience and/or those with higher spatial skills. Additionally, the absence of normative data for the CANTAB limited our ability to determine the extent to which our sample’s cognitive function fell within or outside the normal range.

## Conclusion

The present research identified distinct cortical connectivity differences for cognitive and social CR factors, highlighting the importance of engaging in diverse lifestyle factors in building CR. In our work, high cognitive CR levels were associated with greater eyes-closed, long-range connectivity in low alpha for the occipital region, along with greater eyes-closed, local connectivity in delta for high-CR participants who were men. In contrast, high social CR levels were associated with greater local and long-range connectivity in theta and low alpha frequencies for both eyes-open and eyes-closed recording conditions. For both cognitive and social CR factors, the frequency bands associated with these CR factors in the present research have been linked to cognitive function in older adults in prior research. Finally, we observed significant gender effects in low beta, with greater local and long-range connectivity in women than men, perhaps reflecting slower brain aging in women than men, as has been reported in prior research. In sum, our findings reveal that different CR-building lifestyle factors are associated with distinct effects on the brain’s functional connectivity. These functional connectivity differences could provide a mechanism for improved cognition that is experienced by older adults with high levels of CR.

Although our findings provide evidence for different effects on cortical connectivity stemming from engagement in different lifestyle factors, we believe additional work is needed. First, we explored differences in cortical LLC in conjunction with CR, leaving many electrophysiological measures unexplored. For example, differences in global network topology or activity within specific brain networks (e.g., the default mode network) may play an important role in understanding how CR alters brain function. In addition, our work was limited to resting-state brain activity. Activity during task-directed cognition may offer additional insights to understanding the influence of CR level on cognition. Finally, as we learn more about how CR is acquired, and which lifestyle factors are most important in long-term cognitive health, intervention studies could be conducted to determine if clinical interventions can target deficits in CR and build healthier brains.

## Data Availability Statement

The datasets generated for this study are available on request to the corresponding author.

## Ethics Statement

The studies involving human participants were reviewed and approved by Stockton University’s Institutional Review Board. The patients/participants provided their written informed consent to participate in this study.

## Author Contributions

JF designed the study and wrote the first draft. MA, BD, KC, ED, and OM collected the data. JF, BD, and KC cleaned and processed the EEG data. All authors performed statistical analyses, revised the manuscript, and read and approved the final version.

## Conflict of Interest

The authors declare that the research was conducted in the absence of any commercial or financial relationships that could be construed as a potential conflict of interest.
